# Acute pulmonary edema due to stress cardiomyopathy in a patient with aortic stenosis: a case report

**DOI:** 10.1186/1757-1626-2-9128

**Published:** 2009-12-02

**Authors:** Monika F Bayer

**Affiliations:** 1 PO Box 18736 (at Stanford University) Stanford, California 94309, USA

## Abstract

**Introduction:**

Stress cardiomyopathy is a condition of chest pain, breathlessness, abnormal heart rhythms and sometimes congestive heart failure or shock precipitated by intense mental or physical stress.

**Case presentation:**

A 64-year-old male with a known diagnosis of moderate-to-severe aortic stenosis and advised that valve replacement was not urgent, presented with acute pulmonary edema following extraordinary mental distress. The patient was misdiagnosed as having a "massive heart attack" and died when managed by a traditional protocol for acute myocardial infarction/coronary artery disease, irrespective of his known aortic stenosis.

**Conclusion:**

Intense mental stress poses a considerable risk, particularly to patients with significant aortic stenosis. As described here, it can precipitate acute pulmonary edema. Importantly, effective management of acute pulmonary edema due to stress cardiomyopathy in patients with known aortic stenosis requires its distinction from acute pulmonary edema caused by an acute myocardial infarction. Treatment options include primarily urgent rhythm and/or rate control, as well as cautious vasodilation.

## Introduction

Stress cardiomyopathy or "myocardial stunning" is a condition precipitated by severe physical or emotional stress which was first identified in the 1990s [1]. After an emotional trauma, such as the death of a child or spouse, 10-30 times normal plasma levels of epinephrine, norepinephrine and dopamine, which remained high for several days, have been documented. This toxic increase in circulating catecholamines is thought to "stun" the heart and prevent it from pumping properly [2]. The result is a dangerous reduction in blood flow, chest pain, breathlessness, arrhythmias, and sometimes congestive heart failure or shock. In distinction to an acute myocardial infarction, stress cardiomyopathy per se is generally transient without long-term damage to the heart.

Aortic stenosis (AS) is the most common valvular heart disease [3]. It affects 2-3% of older North Americans and Europeans. The severity of the disease can be difficult to diagnose. It is not known whether exercise testing of asymptomatic patients with severe AS can reliably identify patients most vulnerable to mental stress. In the case reported, the AS was borderline moderate-to-severe and, in the cardiologist's opinion, had not reached a critical stage requiring urgent aortic valve replacement (AVR). Yet, the patient developed acute pulmonary edema in the aftermath of extraordinary distress. Although the current report seems to be the first describing this medical emergency, acute pulmonary edema precipitated by mental stress in patients with AS may not be that rare.

## Case presentation

The case involves a 64-year-old male scientist with a diagnosis of moderate-to-severe aortic stenosis (AS) and mild-to-moderate aortic regurgitation, but no signs and symptoms of congestive heart failure, and no history of myocardial infarction (MI), or coronary artery disease (CAD). The patient had normal blood pressure and was taking no medications. His AS was monitored by serial echocardiograms (Doppler and two-dimensional), the last of which showed a mean transvalvular gradient of 40 mm Hg and a LV end-systolic dimension of 47 mm. The last cardiology evaluation noted that the patient was fairly active but "may be beginning to get mildly symptomatic." He started to notice chest pressure during certain exertion which was promptly relieved by rest and "could be angina." The patient was told that he should not go jogging, that he was near the point where he should consider AVR, but that the AVR "was not urgent". Two months later, the patient became very distressed and angry seeing a family member hurt by outrageous, unlawful conduct. Within about one hour of this stressful event, while engaging in a sedentary activity, he suddenly experienced severe shortness of breath and began to cough up frothy pink sputum. First responders and emergency physicians were informed that the patient had AS and was just extremely upset. In the ambulance, the patient received oxygen, morphine sulfate (10 mg), furosemide (100 mg) and sublingual nitroglycerin. On presentation in the emergency department (ED) about two hours after the extraordinary mental distress began, the patient was alert, had chest rales, a pulse of 123 beats/min, a blood pressure of 121/73 mm Hg, and a respiratory rate of 28-32. His chest X-ray indicated pulmonary edema. During the first 25 minutes in the ED, as he received more nitroglycerin (as paste twice to the chest), another 2 mg morphine sulfate, 2 mg midazolam, and was intubated for ventilation, vigorous diuresis occurred and the patient's initially normal blood pressure plummeted to <70 mm Hg, systolic. At that point, the ECG also showed that the patient had developed atrial fibrillation (AF) with a rapid ventricular rate of 124 beats/min. The patient was started on i.v. dopamine (10 ug/kg/min through most of the next hour). His heart rate increased to148 beats/min, and blood pressure rose to 80-100 mm Hg but then fell again. Several attempts of cardioversion with shocks of 100, 200 and 300 joules, respectively, (7.5 mg valium i.v. for sedation) were begun only 40 minutes after AF was identified, and the patient's condition had worsened considerably. Subsequent administration of digoxin (0.25 mg i.v.) and procainamide (1 g i.v. over 20 min) slowed the ventricular rate to 125 beats/min and raised the blood pressure only briefly to 96/62 mm Hg. Cardioversion attempts (1 × 300 J and 3 × 360 J after 2 × 2.5 mg i.v. additional valium) were continued, but the patient remained in AF with ventricular tachycardia of 120 beats/min and worsening hypotension (BP, 36/30 mm Hg). After a fourth 360 J shock no palpable pulse was noted. After 25 min of CPR including four ampoules of epinephrine, each followed by a further shock of 360 J, which resulted in no rhythm, ventricular fibrillation and agonal rhythm, all resuscitation attempts were terminated. The medical chart summary was that the patient had died from a massive AMI. Yet, cardiac enzymes were negative and the autopsy confirmed aortic stenosis as the cause of death with a bicuspid, heavily calcified aortic valve, left ventricular hypertrophy and moderate dilation, focal pulmonary hemorrhages, primarily mild CAD, no evidence of a recent MI, nor any other major abnormalities.

## Discussion

The very sudden onset of severe dyspnea and cough producing frothy pink sputum about one hour after the patient became extremely upset, as well as all the evidence from his clinical evaluations before and shortly thereafter, strongly indicate that the acute pulmonary edema was precipitated by the preceding emotional trauma and the result of stress cardiomyopathy. Normal cardiac enzymes and the autopsy findings ruled out any AMI.

Irrespective of the patient's known preexisting AS, he was managed primarily with oxygen therapy, morphine sulfate, large doses of both diuretic and nitrates, followed by inotropes. Unfortunately, most of these conventional interventions were not appropriate or beneficial. Current evidence does not support morphine in the treatment of acute pulmonary edema/acute decompensated heart failure [4]. Large doses of nitrites, such as, nitroglycerin paste which can't be properly titrated, should be avoided in advanced AS. Excessive furosemide caused vigorous diuresis potentially contributing to the subsequent precipitous blood pressure fall and intractable hypotension. Individuals who present with a very acute heart failure tend to have it primarily due to a sudden pressure overload causing an abnormal fluid distribution (into lungs), rather than a real volume overload. Aggressive volume depletion leading to hypovolemia can exacerbate a rapid heart rate and promote the development of AF, conditions which in AS almost certainly precipitate hypotension.

Another concern is that electrical cardioversion was delayed for some 40 minutes after AF was positively identified, and medical rhythm/rate control even longer while the patient's condition steadily deteriorated. Prolonged infusion of dopamine, started when the patient became hypotensive, would not be expected to improve his hemodynamic condition. The thickened left ventricle (LV) in AS, which has a low or diminished contractile reserve, can hardly be pushed by inotropes to contract more forcefully, while both the subendocardial perfusion (due to the ventricular tachycardia) and the LV filling pressure (due to prior excessive diuresis and AF) are substantially inadequate. Moreover, when an emotional trauma precipitated the congestive heart failure by flooding the system with catecholamines, the high levels of endogenous epinephrine, norepinephrine and dopamine have been shown to persist for many hours. Thus, more dopamine (with actions that also partially overlap those of epinephrine and norepinephrine), may amplify (not correct) the precipitating factors and exacerbate the heart failure.

According to the general ACC/AHA Guidelines for the Management of Valvular Heart Disease, the cornerstones in the management of acute pulmonary edema in patients with underlying AS should be urgent rhythm and rate control, and vasodilation for decongestion of the lungs [3]. However, when it comes to stress cardiomyopathy and specific interventions to improve symptoms and stabilize such patients for AVR, challenging medical issues remain to be fully resolved. Potential treatment options to consider include the following.

1. In a patient with known AS, paramedics should start respiratory support, but only cautiously administer nitroglycerine, diuretic and, for high blood pressure, angiotensin converting enzyme (ACE) inhibitors.

2. If the pulmonary edema is associated with recent onset AF, or AF develops in the ED, prompt electrical cardioversion to restore normal sinus rhythm and slow the ventricular rate is mandatory. Both AF and a rapid ventricular rate have especially adverse effects in patients with AS. AF can drastically reduce the pre-load, depress pump function and worsen the acute heart failure, since the filling of the hypertrophic LV is up to 40% dependent on proper atrial contractions. Concurrently, AF increases the left atrial and pulmonary venous pressure which exacerbates the pulmonary edema.

3. If the pulmonary edema is associated with severe tachycardia (or AF and electrical cardioversion failed), pharmacologic rate (and rhythm) control is essential in AS. Tachycardia and shorter diastolic perfusion time can cause sustained subendocardial ischemia in a hypertrophied heart with reduced coronary blood flow and limited coronary vasodilator reserve. Subendocardial ischemia in turn can trigger arrhythmias, lead to LV diastolic failure and compromise systolic function. An optimal strategy for the medical control of rapid ventricular rates due to metal stress in AS patients has yet to be defined, but drugs to consider may include the following:

(a) There is a rationale for cautious use of intravenous, short-acting beta-adrenergic blocking agents, such as esmolol and landiolol, if excessive adrenergic stimulation related to mental stress is the suspected mechanism for the tachycardia or tachyarrhythmia.

Esmolol, an ultra short-acting (plasma half life: 9 minutes) and relatively cardioselective beta blocker, has been shown (at 25-300 ug/kg/min) to rapidly slow the heart rate in patients with AF, to effectively relieve chest pain due to acute myocardial ischemia, and to achieve a clinical significant reduction in heart rate (20% or <100 beats per minute) in about 70% of patients, and a conversion to sinus rhythm in about 15%. The principle adverse effect of esmolol is hypotension due to a decrease in cardiac output. But even tachyarrhythmias associated with congestive heart failure, where beta blockers are otherwise contraindicated, may be relatively safely managed with esmolol by starting with a small dose, slowly increasing it while the patient is closely monitored, and decreasing/discontinuing the infusion when complications arise which tends to reverse them [5,6]

Landiolol, a short acting selective beta-1 adrenergic blocker, has been reported to effectively control tachycardia in patients during surgery including one patient with severe aortic stenosis and, in one case, to convert AF to sinus rhythm [7,8]. Beta-blockers with vasodilating effects, such as celiprolol may have the advantage of decreasing the cardiac index the least [9].

(b) Amiodarone (single bolus or short term infusion) effectively treats many cardiac arrhythmias and may be an option for fast heart rate control when other measures fail [10-12]. Although AS patients have generally been excluded from these studies, amiodarone rapidly slowed the ventricular rate and restored normal sinus rhythm in 32% of patients with advanced congestive heart failure, including pulmonary edema and AF. In critically ill patients, who developed AF with a rapid ventricular response and associated fall in systolic blood pressure, amiodarone rapidly and significantly reduced the heart rate with beneficial changes in blood pressure and cardiac output. It has been used in combination with carvedilol (beta1-, beta2-, alpha1-blocker) for rapid reduction of the heart rate in acute AF and severe heart failure [13].

4. For decongestion of the lungs in patients with AS, vasodilators should be preferentially used over diuretics to maintain a sufficiently elevated left ventricular filling pressure and produce an adequate forward stroke volume and cardiac output. Vasodilation with intravenous nitroprusside has been reported to rapidly and significantly increase the cardiac index in critically ill patients with severe AS and congestive heart failure as long as they are not hypotensive [3,14] Particularly, when mental stress and high levels of catecholamines caused vasoconstriction in the coronary and peripheral circulation with a proportional increase in the effective after-load, then nitroprusside may be expected to improve cardiac output by reducing the systemic vascular resistance and correcting the after-load mismatch. To optimize therapy, the nitroprusside must be guided by invasive monitoring and carefully titrated to produce a mean arterial pressure of 60-70 mm Hg.

Administration of both a short acting beta blocker and nitroprusside in combination rather than sequentially may be more effective, if the acute pulmonary edema coexists with acute AF, given the interaction between the two.

## Conclusion

This case illustrates that extraordinary psychological stress can precipitate acute pulmonary edema in patients with advanced AS and, like physical exertion, poses a significant risk to them. These patients and their families need to be warned of this risk to avoid it as much as possible. The differentiation between stress cardiomyopathy and AMI- caused acute pulmonary edema is critically important in patients with known AS for effective emergency care to alleviate symptoms and stabilize them for AVR. Potential treatment options for acute pulmonary edema due to stress cardiomyopathy in such patients include urgent electrical cardioversion, intravenous, short acting beta blockers, or amiodarone for rhythm and/or rate control, as well as cautious vasodilation with intravenous nitroprusside, invasively monitored to decongest the lungs and improve LV performance. By contrast, aggressive diuresis and preload reduction must be avoided to prevent intractable hypotension and hypoperfusion. Inotropes like dopamine are not indicated after emotional stress and catecholamine surge.

## Abbreviations

AS: aortic stenosis; AVR: aortic valve replacement; AF: atrial fibrillation; MI: myocardial infarction; CAD: coronary artery disease; ED: emergency department; LV: left ventricle.

## Consent

Written informed consent was obtained from the widow for the purpose of publication of the manuscript. A copy of the written consent is available for review by the editor-in-Chief of this journal.

## Competing interests

The author declares that she has no competing interests.
